# A Balance between the Activities of Chloroplasts and Mitochondria Is Crucial for Optimal Plant Growth

**DOI:** 10.3390/antiox10060935

**Published:** 2021-06-09

**Authors:** Zhou Xu, Renshan Zhang, Meijing Yang, Yee-Song Law, Feng Sun, Ngai Lung Hon, Sai Ming Ngai, Boon Leong Lim

**Affiliations:** 1 School of Biological Sciences, University of Hong Kong, Pokfulam, Hong Kong, China; jodiexu@connect.hku.hk (Z.X.); rs_zhang@fudan.edu.cn (R.Z.); celine30@connect.hku.hk (M.Y.); yeesong0210@gmail.com (Y.-S.L.); epusun@sdu.edu.cn (F.S.); 2 School of Life Sciences, The Chinese University of Hong Kong, Shatin, Hong Kong, China; gnulecneics@gmail.com (N.L.H.); smngai@cuhk.edu.hk (S.M.N.); 3 State Key Laboratory of Agrobiotechnology, The Chinese University of Hong Kong, Shatin, Hong Kong, China

**Keywords:** ATP, AtPAP2, chloroplasts, mitochondria, redox

## Abstract

Energy metabolism in plant cells requires a balance between the activities of chloroplasts and mitochondria, as they are the producers and consumers of carbohydrates and reducing equivalents, respectively. Recently, we showed that the overexpression of *Arabidopsis thaliana* purple acid phosphatase 2 (AtPAP2), a phosphatase dually anchored on the outer membranes of chloroplasts and mitochondria, can boost the plant growth and seed yield of *Arabidopsis thaliana* by coordinating the activities of both organelles. However, when AtPAP2 is solely overexpressed in chloroplasts, the growth-promoting effects are less optimal, indicating that active mitochondria are required for dissipating excess reducing equivalents from chloroplasts to maintain the optimal growth of plants. It is even more detrimental to plant productivity when AtPAP2 is solely overexpressed in mitochondria. Although these lines contain high level of adenosine triphosphate (ATP), they exhibit low leaf sucrose, low seed yield, and early senescence. These transgenic lines can be useful tools for studying how hyperactive chloroplasts or mitochondria affect the physiology of their counterparts and how they modify cellular metabolism and plant physiology.

## 1. Introduction

The crosstalk between chloroplastic photosynthesis and mitochondrial respiration has long been discussed, given that they share important metabolites that serve as products and substrates, respectively, in these two organelles [1]. While chloroplasts are the major generators of reducing equivalents, mitochondria are the major organelles that dissipate reducing equivalents in plant cells. In chloroplasts, the light-driven photosystems generate phosphorylating power (adenosine triphosphate, ATP) and reducing equivalents (nicotinamide adenine dinucleotide phosphate, NADPH) before the chemical energy carried by these molecules is transferred to carbohydrates through CO_2_ assimilation [2]. While carbon fixation requires an NADPH/ATP ratio of 0.67, the linear electron flow (LEF) of the photosystem generates an NADPH/ATP ratio of 0.78 [3], and the surplus in reducing equivalents has to be consumed or dissipated to regenerate NADP^+^, the electron acceptor of the LEF [4]. Once the chloroplast is overactivated by excess light or other triggers, reducing equivalents will accumulate and cause overreduction of the photosynthetic electronic transport chain (ETC), leading to reactive oxygen species (ROS) generation and photodamage [5,6,7]. Plants control overreduction and oxidative stresses by multiple mechanisms, such as the mobilization of ROS scavengers, the activation of chloroplast-located alternative electron pathways, and the utilization of excess reducing equivalents for nitrogen and sulfur assimilation [8].

Excess reducing equivalents can also be exported from the chloroplasts via the malate–oxaloacetate (OAA) shuttle [9]. In green algae and diatoms, excess reducing equivalents from chloroplasts can be redirected to mitochondria to drive ATP synthesis and to maintain the redox balance in chloroplasts [10,11,12]. However, in C3 plants, photorespiration generates a large amount of reducing equivalents in mitochondria via glycine decarboxylation, which exceeds the NADH-dissipating capacity of mitochondria, and the surplus NADH is indirectly exported from the mitochondria to the cytosol through the malate–OAA shuttle [13]. Hence, in *Arabidopsis thaliana*, while mitochondria import malate in the dark, they export it in the light. Malate therefore serves as a reservoir of surplus reducing equivalents during photosynthesis [13]; its level increases during photosynthesis and decreases in the dark [14].

*Arabidopsis thaliana* purple acid phosphatase 2 (AtPAP2) is a phosphatase dually anchored on the outer membranes of chloroplasts and mitochondria via its hydrophobic C-terminal motif, with its phosphatase domain facing the cytosol [15,16]. AtPAP2 plays a role in protein import into these two organelles, possibly by the removal of phosphate groups from phosphorylated transit peptides [17] or presequences of imported proteins [18,19]. The overexpression (OE) of AtPAP2 in Arabidopsis results in faster plant growth and ~50% higher seed yield than the wild type (WT) [16]. The OE lines were shown to contain a higher sugar content [16] and higher ATP and adenosine diphosphate (ADP) levels in leaves than the WT [20]. 

The overexpression of AtPAP2 in chloroplasts alters the photosystem I/photosystem II (PSI/PSII) ratio and composition of the photosystems, enhances the LEF, and increases the capacity of Calvin–Benson–Bassham (CBB) enzymes. In addition, the overexpression of AtPAP2 in mitochondria alters the composition of the respiratory chain and the abundance of various tricarboxylic acid cycle (TCA) enzymes, thereby enhancing mitochondrial reductant-dissipating capacity [4]. The ability of mitochondria to dissipate reducing equivalents is important for relieving the buildup of surplus reducing equivalents in stroma, an excess of which will limit the LEF due to an insufficient supply of NADP^+^. A higher consumption of reducing equivalents by mitochondria not only generates more ATP for sucrose synthesis, but also decreases the export of reducing equivalents from the mitochondria and thus the overall redox status of the cells. Efficient cooperation between chloroplasts and mitochondria eventually boosts the growth and seed yield of OE lines [4]. In *Camelina sativa*, overexpression lines of AtPAP2 also bolt earlier and generate higher seed yield than the WT [21].

Here, we created transgenic lines that solely overexpress AtPAP2 in either chloroplasts or mitochondria. These OE lines exhibit specific growth phenotypes and are unique tools to illustrate how hyperactive chloroplasts or mitochondria could affect plant physiology.

## 2. Materials and Methods

### 2.1. Plant Lines and Growth Conditions

Arabidopsis lines used in this study included the wild-type *Arabidopsis thaliana* ecotype Columbia-0 (WT), the dually targeted AtPAP2 overexpression line (OE7, OE21) [4,16], and lines with AtPAP2 solely overexpressed in mitochondria (P2TOM) or chloroplasts (P2TOC) generated in this study. Homologous T3 seeds were used. Seedlings were grown by two methods: (1) Chilled seeds were surface-sterilized with 20% (*v/v*) bleach for 15 min, washed with sterile water, and plated onto Murashige and Skoog (MS) medium supplemented with 2% (*w/v*) sucrose. Then, 8-day-old seedlings with similar sizes were transplanted to soil. (2) Seeds were chilled in water for 3 days and directly sown in the soil. All plants were grown under a long-day regime (16 h light (22 °C)/8 h dark (18 °C)) with 120–150 µmol m^−2^ s^−1^ of light density.

### 2.2. Cloning of C-tails into Enhanced Green Fluorescent (EGFP) Constructs

The C-terminal motifs of AtToc33 (At1g02280) and AtTom20-3 (At3g27080) were amplified from cDNA of WT Arabidopsis using the primer pairs TOC33GFPF and TOC33GFPR, and TOM20GFPF and TOM20GFPR (Supplementary Table S1). Polymerase chain reaction (PCR)-amplified cDNA fragments were cloned into the pBI221 backbone modified to contain EGFP under the CaMV35S (Cauliflower Mosaic Virus 35S) promoter for transient expression in Arabidopsis protoplasts.

### 2.3. Generation of Transgenic Line of P2TOM and P2TOC in Arabidopsis

The coding sequences (CDSs) of the C-termini of AtTom20-3 and AtToc33 were merged with those of the phosphatase domain of AtPAP2 via overlapping PCR technique to create the constructs of P2TOM and P2TOC, respectively. The two constructs were then cloned downstream of a CaMV 35S promotor of the pCXSN vector for further Agrobacteria and Arabidopsis transformation. All primers used are listed in Table S1.

### 2.4. Sodium Dodecyl Sulfate–Polyacrylamide Gel Electrophoresis (SDS-PAGE)/Western Blotting

In the next step, 25 µg chloroplast or mitochondrial proteins or 30 µg leaf proteins was separated by sodium dodecyl sulfate–polyacrylamide gel electrophoresis (SDS-PAGE) and transferred to nitrocellulose blotting membrane (GE Healthcare, Hong Kong, China) as described previously [22,23]. Anti-AtPAP2 antibodies were generated previously [16].

### 2.5. Isolation of Mitochondria from Arabidopsis Leaves Using Continuous Gradient Centrifugation

Arabidopsis mitochondria were isolated as previously described with modifications [24,25]. All procedures were performed at 4 °C, including sample harvest, solution, and centrifugation. Around 20 g of Arabidopsis leaves was sampled and ground by mortar and pestle while chilled in a 50 mL grinding buffer containing 0.3 M sucrose, 25 mM tetrasodium pyrophosphate, 2 mM EDTA, 10 mM KH_2_PO_4_, 1% (*w/v*) PVP-40, 1% (*w/v*) BSA at pH 7.5, 20 mM ascorbate, and 20mM L-cystein (added on the day of use). After 2 min of continuous grinding, the homogenate was filtered through a double layer of Miracloth and rinsed again with 50 mL of grinding buffer. The combined homogenate was centrifuged at 2450× *g* for 5 min. The supernatant was transferred to a 50 mL polycarbonate centrifuge tube and centrifuged at 17,400× *g* for 20 min. The resulting supernatant was discarded, and the pellet was resuspended in wash buffer (0.3 M sucrose, 10 mM TES, 0.1% (*w/v*) BSA, pH 7.5). This was further loaded onto Percoll^TM^ (GE Healthcare) continuous gradient containing 0 to 4.4% (*w/v*) PVP. After centrifugation at 40,000× *g* for 40 min, mitochondria were concentrated in a pale-yellow band near the bottom of the tube. The layer of mitochondria was then transferred to a new polycarbonate centrifuge tube with BSA-free wash buffer and centrifuged at 2450× *g* for 15 min. After 3–4 washing steps, the mitochondrial pellet was resuspended in the wash buffer without BSA. The amount of mitochondrial protein was determined by Bradford protein assay (Bio-Rad, USA). The mitochondria were stored at −80 ℃ until use.

### 2.6. Isolation of Chloroplast from Arabidopsis Leaves Using Discontinuous Gradient Centrifugation

The method of Arabidopsis chloroplast isolation was modified from previous reports [26,27]. During isolation, all plants, buffers, and equipment were kept at 4 ℃. Samples of 10 g of Arabidopsis leaves were cut and homogenized 9 times in 10 s bursts with 5 s pauses in between. This was performed using a Polytron (T 25 digital ULTRA-TURRAX^®^, Ika Works Inc., Wilmington, USA) at a speed of 19,000 rpm with a 13 mm diameter rotor in 40 mL homogenization buffer (0.3 M sorbitol, 5 mM EGTA, 5 mM EDTA, 10 mM NaHCO_3_, 0.1% (*w/v*) BSA, 1 mM DTT, and 20 mM Tricine, pH 8.4) in a 200 mL centrifuge bottle. The homogenate was filtered through a double layer of Miracloth (Merck, Darmstadt, Germany) twice by rehomogenizing the debris retained in the Miracloth in 40 mL homogenization buffer. The crude chloroplasts were sedimented by centrifugation at 1000× *g* for 5 min (brake off), and the pellets were resuspended in 1 mL sorbitol buffer (0.3 M sorbitol, 5 mM MgCl_2_, 2.5 mM EDTA, 10 mM NaHCO_3_, 0.1% BSA (*w/v*), 20 mM HEPES, pH 7.6) using a paintbrush. The mixture was then loaded onto a discontinuous Percoll^TM^ (GE Healthcare, Hong Kong, China) gradient containing 0.3 M sorbitol, 5 mM MgCl_2_, 2.5 mM EDTA, 10 mM NaHCO_3_, 0.1% (*w/v*) BSA, 20 mM HEPES, pH 7.6, with 40% (*v/v*) Percoll^TM^ in the top layer (15 mL) and 100% (*v/v*) Percoll^TM^ in the bottom layer (5 mL). After centrifugation at 2000× *g* for 10 min using a swing-bucket rotor with brake off, the purified chloroplasts in the interphase between the layers of 40 and 100% Percoll^TM^ (GE Healthcare) were transferred to a Falcon tube. The collected chloroplasts were then washed twice with sorbitol buffer without BSA by centrifuging (brake off) at 1000× *g* for 2 min. Chloroplast protein content was determined by Bradford protein assay (Bio-Rad, Hong Kong, China). The chloroplasts were then stored at −80 ℃ until use.

### 2.7. Protoplast Transient Expression Assay

Arabidopsis mesophyll protoplast preparation and transfection were carried out according to a method described previously [28]. Protoplasts isolated from 4-week-old rosettes were transfected with plasmids and cultured for 10 h for protein expression. Then, 0.1 mL (10^5^) of protoplasts was transfected with 8 μg of DNA. Plasmids for transient expression analysis were extracted using a Hipure EF Plasmid Kit (Magen, Guangzhou, China). To mark mitochondria in protoplasts, protoplasts were incubated with culture medium containing 100 nM MitoTracker (Thermo Fisher Scientific, Hong Kong, China) for 30 min, and the MitoTracker was removed before confocal observation by washing the protoplasts with culture medium twice.

### 2.8. Enzymatic Assays

The TCA enzyme activity of mitochondrial proteins was measured as described in the previous study [29]. Enzyme assays of fructose 1,6-bisphosphate aldolase (FBA) [30] and NADP^+^–glyceraldehyde-3-phosphate dehydrogenase (GAPDH) [31] were performed on the chloroplast proteins. All enzyme assays were performed at 25 ℃ using a Multiskan^TM^ GO Microplate Spectrophotometer (Thermo Fisher Scientific, Hong Kong, China).

### 2.9. Chlorophyll Fluorescence Measurement

Twenty-day-old Arabidopsis plants were sampled directly on a 6-pot tray and adapted in the dark for more than 1 h. Chlorophyll fluorescence was then measured by the IMAGING-PAM M-Series Maxi Version (Walz Company, Effeltrich, Germany). Maximum fluorescence (Fm) and initial fluorescence (Fo) were measured intermittently based on 40 s intervals. After the dark treatment, a series of light intensities (0, 81, 145, 186, 281, 335, 461, 701, and 926 μmol m^−2^ s^−1^) was used to illuminate the leaves at a duration of 3 min each. At the end of the 3 min illumination, a saturation pulse was applied. ImagingWin software was used for analyzing the values of the photosynthetic electron transport rate (ETR), photochemical quenching (qP), non-photochemical quenching (NPQ), and efficiency of photosystem II (Y(II)) against light intensity using the following equations (1)–(4) [32]:

ETR = 0.5 × 0.84 × ((Fm’- Fs) /Fm’) × light intensity (μmol photons m^−2^ s^−1^), (1)

qP = (Fm’− Fs)/ (Fm’− Fo),(2)

NPQ = (Fm − Fm’)/ Fm’,(3)

Y(II) = (Fm’ − Fs)/ Fm’(4)

### 2.10. Imaging of Leaf Mitochondrial Superoxide (O_2_^●−^)

MitoSOX^TM^ Red mitochondrial O_2_^●−^ indicator (#M36008, Thermo Fisher Scientific, Hong Kong, China) is a live-cell permeant dye selectively targeted to mitochondria, where it is oxidized by superoxide and releases red fluorescence [33]. The imaging method was slightly modified from a previous paper [34]. The youngest, most fully expanded leaves of 20-day-old Arabidopsis were incubated in 5 µM MitoSOX^TM^ Red reagent (10 mM KH_2_PO_4_, pH 7.4, with KOH) with vacuum infiltration for 1 h in the dark at room temperature, after the lower epidermis of the leaves was peeled off with a razor blade. The leaf segments were then rinsed thrice with the wash solution (0.5× MS medium, pH 5.8, with KOH) in the dark. The leaf was then mounted onto a microscope slide, with the wounded side facing the slide cover. The mesophyll cell layer was briefly (~1 min) illuminated by white light (~50 µmol m^−2^ s^−1^) for focusing and then examined directly with a Zeiss LSM710 confocal microscope (Carl Zeiss, Jenna, Germany) with appropriate laser apparatus and detection settings (MitoSOX Red, 488/585–615). A 63× oil-immersion lens in multi-track mode with line switching was used to perform the imaging. All images were captured under similar settings.

### 2.11. Luciferase ATP/ADP Measurement

Around 100 mg of 20-day-old Arabidopsis leaves was sampled and ground into fine powder in liquid N_2_. Then, 500 µL of 2.3% (*v/v*) TCA was added to each sample. After centrifuging at 2000× *g* for 15 min at 4 °C, the supernatant was transferred to a new tube and the pH was adjusted to 7.0 using a few drops of 2.5 M K_2_CO_3_ [35]. Each 100 µL of ATP extract was diluted 10-fold and divided into two portions, one for direct ATP measurement and the other for measurement of total ATP, including those converted from ADP. A total of 50 µL of the ADP buffer (15 mM Tricine pH 7.5, 2 mM MgCl_2_, 4 mM KCl, 1 mM phosphoenolpyruvate, and 0.8 µg/mL of pyruvate kinase) was added to 200 µL of sample extract to convert ADP to ATP. After 30 min of incubation at 30 °C, the sample was boiled at 100 °C for 3 min and cooled down at room temperature. The ATP measurement was carried out using an ATP Bioluminescent Assay Kit (Sigma with a VICTOR Multilabel Plate Reader (PerkinElmer, Wellesley, USA)) [36]. Thus, the ADP content could be calculated by subtracting the ATP level of the extract before pyruvate kinase conversion from the total ATP level after conversion.

### 2.12. Gas Chromatography-Mass Spectrometry (GC-MS/MS) Measurement

Metabolite profiling of Arabidopsis leaves by GC-MS was carried out on an Agilent 7890B GC system equipped with Agilent 5977B Mass Selective Detector (MSD), which uses electroionization (EI) as the ionization source [37]. The GC-MS raw data were collected and first analyzed by enhanced data analysis in the MSD ChemStation software (Agilent, Santa Clara, CA, USA). The original AIA format of GC-MS data was uploaded to the XCMS website (https://xcmsonline.scripps.edu/landing_page.php?pgcontent=mainPage, accessed date 24 October 2018) via a secure SSL connection to process direct comparisons of total ion current (TIC) chromatograms. The library installed in the ChemStation software was used to identify metabolites from GC-MS chromatograms. The intensity of metabolites was calculated from the total peak area, baseline width, and normalization to the quality control (QC) sample and the error rate of internal standard (ribitol).

## 3. Results

### 3.1. Growth Phenotypes of P2TOM and P2TOC Lines

**Figure 1:**
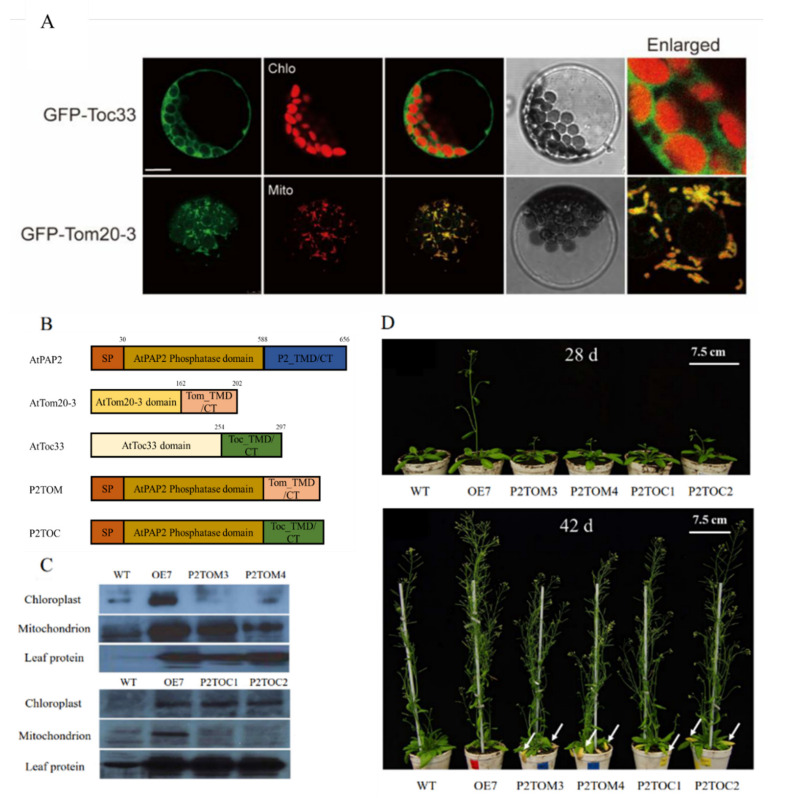
Growth phenotypes of AtPAP2 overexpression lines. (**A**) Transient expression of GFP-Toc33 and GFP-Tom20-3 in Arabidopsis protoplasts. Protoplasts were transformed with constructs of AtToc33 and AtTom20-3 fused with GFP at their N-termini, respectively. Representative confocal images of GFP fluorescence are shown. Chlo, chloroplast auto-fluorescence; Mito, mitochondria marked by Mitotracker. Scale bar, 10 µm. (**B**) Construction of P2TOC and P2TOM overexpression vectors that replaced C-terminal motif of AtPAP2 with that of AtToc33 or AtTom20-3. (**C**) Confirmation of AtPAP2 expression in chloroplasts and/or mitochondria and total leaf protein of transgenic lines by Western blotting analysis using anti-AtPAP2 antibodies. (**D**) 28- and 42-day-old plants under a long-day (16 h/8 h) regime. The yellow necrotic spots were indicated by white arrows. Scale bar, 7.5 cm.

Many preprotein receptors of the translocons on the outer membranes of the two organelles are tail-anchored (TA) proteins, which harbor a single transmembrane domain (TMD) at the C-termini of the preprotein receptors, followed by a short hydrophilic tail [16,38]. In protoplast experiments, the C-terminal motifs of AtToc33 and AtTom20-3 were able to target GFP to the outer membranes of chloroplasts and mitochondria, respectively (Figure 1A). In order to specifically target the phosphatase domain of AtPAP2 solely to the outer membranes of chloroplasts or mitochondria, its native C-terminal motif was replaced by the C-terminal motifs of AtToc33 and AtTom20-3, respectively (Figure 1B). After stable transformation, two homozygous lines from each binary construct were randomly selected for examination: P2TOC1 and P2TOC2 lines of the P2TOC construct, and P2TOM3 and P2TOM4 lines of the P2TOM construct. To verify whether AtPAP2 is solely overexpressed in the chloroplasts of P2TOC lines or the mitochondria of P2TOM lines, protein extracts from isolated chloroplasts, mitochondria, and rosette leaves of T3 homozygous OE7, P2TOC, P2TOM, and WT lines were probed with anti-AtPAP2 antibodies. The OE7 and WT lines were loaded as controls. The two P2TOM lines showed a higher abundance of AtPAP2 in mitochondria than WT, but their levels in chloroplasts were comparable (Figure 1C). In contrast, the two P2TOC lines only contained the WT level of AtPAP2 in mitochondria, but a higher abundance in chloroplasts (Figure 1C). The AtPAP2 levels in the leaf protein of P2TOM and P2TOC were consistent with the levels in P2TOM mitochondria and P2TOC chloroplasts, respectively (Figure 1C). As reported in a previous study [16], the OE7 line contains higher levels of AtPAP2 in the chloroplasts, mitochondria, and total leaf protein extract.

The OE7, P2TOC, P2TOM, and WT lines were grown under long-day conditions. All AtPAP2 overexpression lines, including OE7, P2TOC, and P2TOM, bolted earlier than WT (Figure 1C and Table 1), and the number of rosette leaves on the first day of appearance of inflorescence (NRF) was significantly lower in the AtPAP2 overexpression lines (Table 1). This indicates that AtPAP2 overexpression, in either chloroplasts or mitochondria or both organelles, can accelerate flowering in Arabidopsis. Furthermore, the P2TOC and P2TOM lines had yellowed and necrotic spots on their rosette leaves at day 42 (Figure 1D), indicating that AtPAP2 overexpression in either chloroplasts or mitochondria led to early senescence. Compared to WT, the OE7 (+40%) and P2TOC (+20–24%) lines produced significantly higher seed yields, whereas both P2TOM lines (−74%) had lower seed yield at maturity (Table 1).

### 3.2. Mitochondrial Activity of OE7, P2TOM, and P2TOC Lines was Higher Than WT

**Figure 2:**
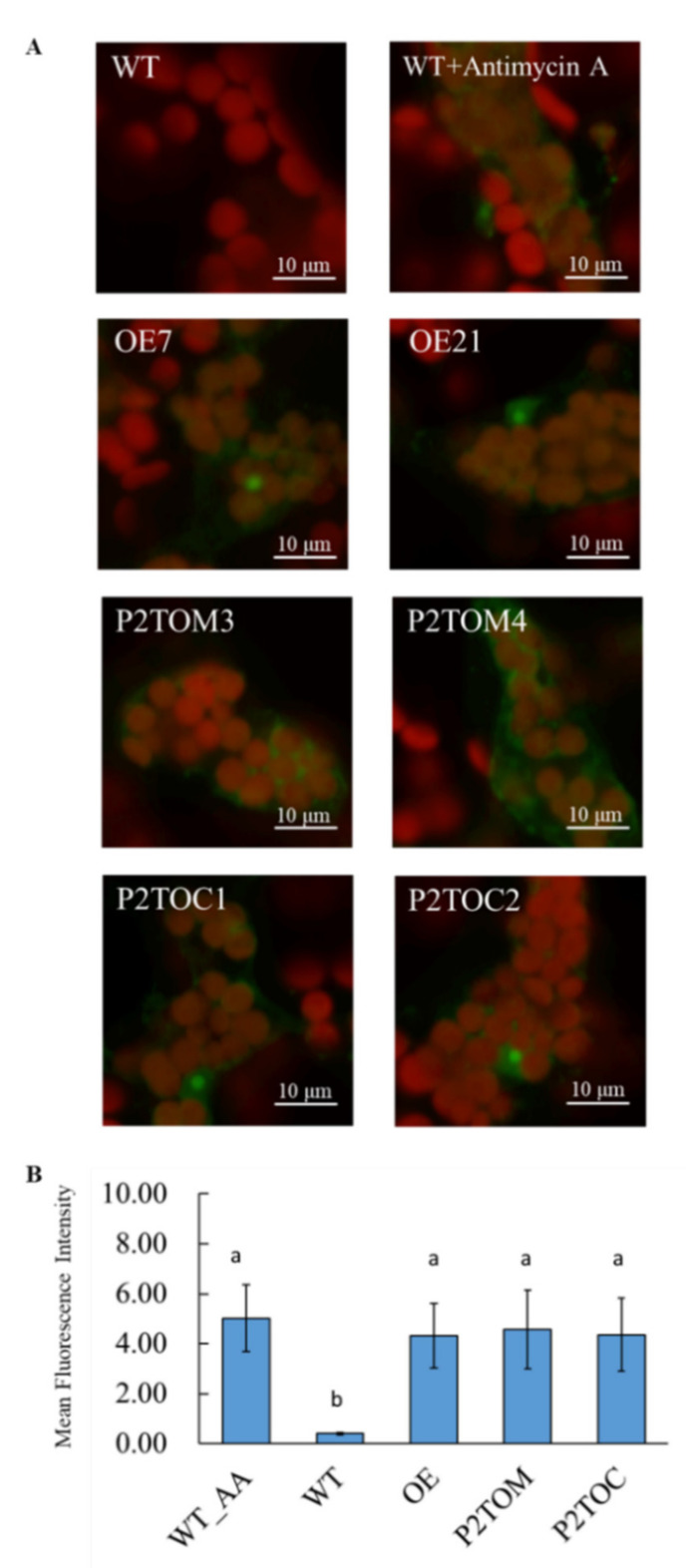
Confocal images of MitoSOX Red in WT and AtPAP2 OE lines. (**A**) MitoSOX Red probe was applied to peeled lower epidermis leaves from 20-day-old plants. Fluorescence of MitoSOX Red (565–615 nm) and autofluorescence of chloroplasts (>650 nm) are shown in green and red pseudocolors, respectively. WT treated with antimycin A and untreated WT were positive and negative controls, respectively. White scale bar indicates 10 µm. (**B**) Mean fluorescence intensity of WT (treated with AA), WT, OE7, P2TOM, and P2TOC lines was analyzed by one-way ANOVA with Tukey’s HSD test using PASW Statistics 18 software. Different letters (a, b) above each bar (*n* = 4) represent significant differences (*p* < 0.05).

The P2TOC1, P2TOM4, and OE7 lines were selected for further analysis. Chloroplasts and mitochondria were isolated from 20-day-old plants and the capacity of enzymes involved in the TCA cycle was compared with that of WT (Table 2). The capacity of many TCA enzymes was upregulated in the mitochondria of P2TOM, P2TOC, and OE7 lines (Table 2). In plant cells, mitochondrial ETC is the major site for ROS generation [39]. When electron carriers are over-reduced, excess electrons in the ETC leak out, reducing O_2_ to form ROS [40]. To visualize the ROS production in mitochondria in vivo, a cell-permeable probe, MitoSOX Red, which targets mitochondria selectively, was applied to Arabidopsis leaves [34,41]. As a positive control, WT leaves were treated with the complex III inhibitor antimycin A (AA) for 1 h before incubation with MitoSOX Red (Figure 2). Without AA treatment, WT leaves did not show any ROS signal in the emission range of 565–615 nm (Figure 2). Under the same settings, P2TOM, P2TOC, and OE7 lines displayed higher concentrations of O_2_^●−^ compared to WT, and their signal intensities were similar to the positive control (Figure 2). The mean fluorescence intensity of four biological replicates was analyzed by ImageJ software (Figure 2B). These results imply that the mitochondrial ETC of P2TOM, P2TOC, and OE7 lines is overloaded with electrons, leading to electron leakage and further ROS generation.

### 3.3. Chloroplastic Activity was Higher in OE7 and P2TOC but Lower in P2TOM Than in WT

**Figure 3:**
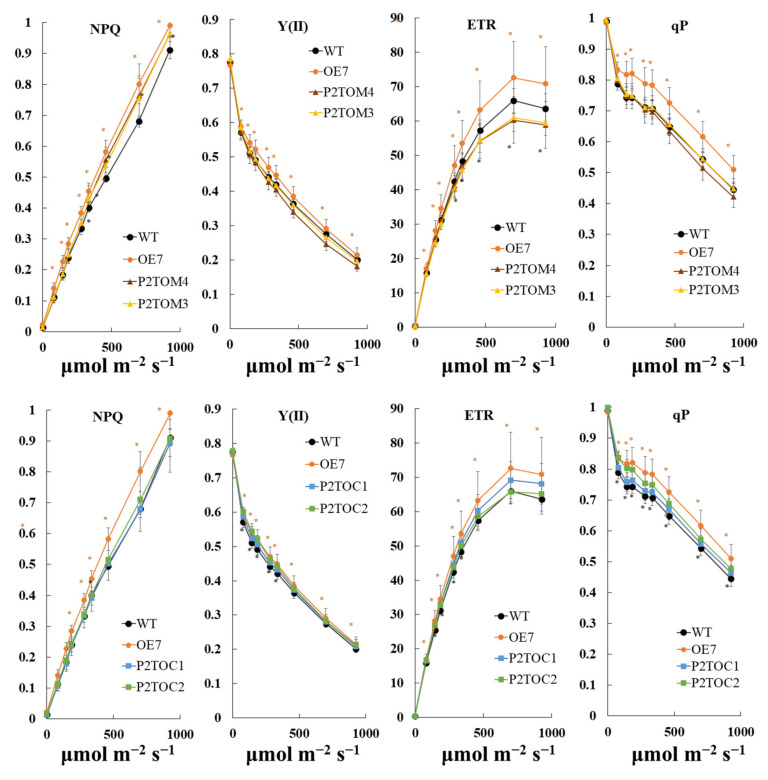
In vivo measurement of electron transport activity. Light density-dependent NPQ, Y(II), ETR, and qP of WT (black circles), OE7 (orange circles), P2TOC1 (blue squares), P2TOC2 (green squares), P2TOM3 (yellow triangles), and P2TOM4 (brown triangles) lines were acquired by MAXI-PAM. Twenty-day-old plants were dark-adapted for 1 h before measurement at 9 light densities (0, 81, 145, 186, 281, 335, 461, 701, and 926 μmol m^−2^ s^−1^) with a duration of 3 min each. Values are mean ± SD (*n* > 10 per line). Asterisks show significant differences between WT and the TOC/TOM lines according to t-test, * *p* < 0.05 (orange: OE7, black: P2TOM/P2TOC lines).

To study the photosynthetic performance of the AtPAP2 overexpression lines, chlorophyll fluorescence analysis was applied to 20-day-old Arabidopsis leaves using IMAGING-PAM. The data demonstrated that OE7 had higher NPQ, Y(II), ETR, and qP under nine light intensities, while P2TOC scored higher in those parameters only when the light intensity was lower than 461 μmol m^−2^ s^−1^ (Figure 3). For the P2TOM line, a lower ETR value was observed compared to WT, which implies that the P2TOM line was less efficient in carbon assimilation (Figure 3). The Y(II) and qP of P2TOM were lower compared to WT, but the differences were not significant (Figure 3). Overall, ETR efficiency decreased in the order of OE7, P2TOC, WT, and P2TOM. The CBB cycle in chloroplasts converts CO_2_ and light energy harvested by the photosystems into carbohydrates. In terms of the capacity of CBB enzymes, the capacity of NADP^+^-GAPDH and malate dehydrogenase (MDH) was significantly higher in the P2TOC chloroplasts compared to WT by 12 and 23%, respectively. OE7 displayed increased enzyme capacity for the CBB cycle as well. For P2TOM chloroplasts, the capacity of FBA and GAPDH in the CBB cycle was lower compared to WT by 13 and 10%, respectively (Table 2).

### 3.4. Impacts of AtPAP2 Overexpression on the Plant Energy Status

**Figure 4:**
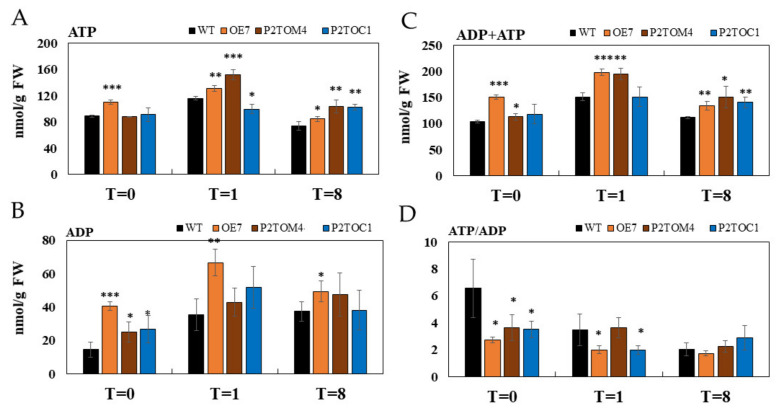
Altered adenosine phosphate levels in leaves of transgenic lines. (**A**) ATP content, (**B**) ADP content, (**C**) ADP + ATP content, and (**D**) ATP/ADP ratio. T = 0, 1, 8 represent end of night, 1 h of illumination after darkness, and 8 h of illumination (midday). Data were calculated by means of three biological replicates. Significant differences between transgenic lines and WT are indicated by asterisks (* *p* < 0.05, ** *p* < 0.01, *** *p* < 0.001).

At the middle of the day (T = 8), all AtPAP2 overexpression lines had significantly higher ATP levels compared to WT, but only OE7 had a higher ATP level than WT at the end of the night. Interestingly, the leaf ATP levels were in the following order at 1 h after illumination: P2TOM > OE7 > WT > P2TOC (Figure 4A). At the end of the night (T = 0), all AtPAP2 overexpression lines had significantly higher ADP levels compared to WT, but at the other two time points, only OE7 had a significantly higher ADP level than WT (Figure 4B). In terms of the sum of ATP and ADP, OE7 had a higher content than WT at all three time points, whereas P2TOC and P2TOM had higher ATP + ADP than WT at one and two time points, respectively (Figure 4C). Due to the higher ADP level in the three OE lines than WT at the end of the night, the ATP/ADP ratio was significantly lower for the three OE lines (Figure 4D). Upon illumination, the ATP/ADP ratio of WT dropped until the ratios of the four lines became indifferent at the middle of the day (Figure 4D). 

### 3.5. Impact of AtPAP2 Overexpression on Leaf Metabolites

**Figure 5:**
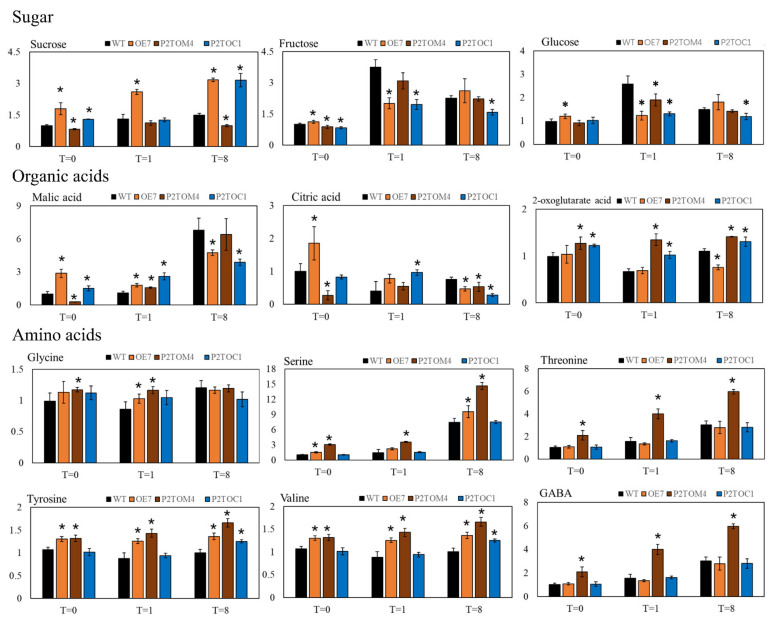
Metabolomic data of 20-day-old Arabidopsis leaves collected at T = 0, 1, and 8 h after illumination. Data were measured by GC-MS and normalized with data obtained from WT at T = 0. Around 150 mg FW was used in extraction and ribitol was used as internal standard. Values represented by bars are means normalized to mean data of WT at T = 0. Asterisks represent significant differences in OE7 or P2TOM or P2TOC lines compared with WT (black bar) at corresponding time points (T = 0, 1, 8), *n* = 4–6, * *p*-value < 0.05. GABA, gamma-aminobutyric acid.

The leaf contents of 12 metabolites, including three sugars, three organic acids, and six amino acids, were measured. Regarding sucrose, the major sugar supplied from mesophyll to sink tissues to drive plant growth, the OE7 line contained a significantly higher sucrose content than the WT at all three time points. The P2TOC line had a sucrose level comparable to OE7 only at T = 8, and at T = 1 it was similar to that of WT. At T = 0, the sucrose content of P2TOC was higher than that of WT but lower than that of OE7 (Figure 5). In contrast, the sucrose content of P2TOM was significantly lower than that of WT at T = 0 and T = 8. In general, the sucrose levels were in the order of OE7 > P2TOC > WT > P2TOM, which correlated with the seed yields at maturity (Table 1). The fructose and glucose contents were significantly higher in OE7 than WT at T = 0, but significantly lower in OE7 and P2TOC than WT after 1 h of illumination. After 8 h of illumination, P2TOC contained less fructose and glucose than OE7, WT, and P2TOM. These changes show that carbon metabolism in the leaf is greatly impacted by altered chloroplast and/or mitochondrial activities due to the overexpression of AtPAP2 on these organelles.

In addition to sugars, remarkable variations among three organic acids in the TCA cycle, malate, citrate, and 2-oxoglutarate (2-OG), were also observed (Figure 5). Generally, malate levels increase gradually with the duration of illumination, as malate is the reservoir of surplus reducing equivalents generated by chloroplasts (photosynthesis) and mitochondria (photorespiration). At the end of the night, after the consumption of malate during prolonged darkness, the malate content was in the following order: OE7 > P2TOC > WT > P2TOM (Figure 5). After 1 h of illumination, the malate levels of the three AtPAP2 overexpression lines were higher compared to WT. In contrast, in the middle of the day (T = 8), while OE7 and P2TOC contained the highest malate content of the three time points, the levels were significantly lower than those of WT and P2TOM (Figure 5). One explanation for this observation is that during prolonged illumination, OE7 and P2TOC fixed more sucrose and consumed more reducing equivalents in CO_2_ fixation than the other two lines. Regarding citrate, the level was significantly higher in OE7 and lower in P2TOM than in WT at the end of the night. After 1 h of illumination, P2TOC had significantly higher amounts of citrate than the other three lines. After prolonged illumination, the citrate level was significantly lower in all three AtPAP2 overexpression lines than in WT. For 2-OG, it was significantly higher in P2TOC and P2TOM at all three time points, but significantly lower in OE7 at the middle of the day. 

Interestingly, the amino acid content in the P2TOM line changed noticeably, while it was relatively stable in the P2TOC line. P2TOC only had significantly higher tyrosine and valine at the middle of the day (T = 8). Specifically, serine levels significantly increased in OE7 and P2TOM, possibly due to a higher rate of photorespiration (Figure 5).

## 4. Discussion

Chloroplasts and mitochondria are the main powerhouses of plant cells, and they cooperatively produce adequate ATP to meet the demands of various metabolic processes [42]. AtPAP2, which is dually anchored on the outer membranes of chloroplasts and mitochondria via its C-terminal hydrophobic motif, modulates the importation of nucleus-encoded proteins into chloroplasts and mitochondria [15,17,18]. Arabidopsis with overexpression of AtPAP2 was reported to grow faster and produce higher biomass and seed yield [16]. OE7 chloroplasts exhibited higher LEF and carbon fixation rate and had a high capacity of CBB enzymes [4], while OE7 mitochondria had a high capacity of TCA enzymes and exhibited a higher capacity of consuming reducing equivalents. The combined actions of hyperactive chloroplasts and mitochondria in the OE7 line streamline the production and consumption of reducing equivalents of these organelles and balance the ATP/NADPH ratio during photosynthesis [4]. For example, a higher consumption of reducing equivalents by mitochondria can relieve the accumulation of NADPH in chloroplasts and regenerate NADP^+^, the major electron acceptor of LEF [13]. The higher output of 3C compounds from OE7 chloroplasts and of ATP from OE7 mitochondria results in higher sucrose synthesis. This enables the fast growth rate and higher seed yield of the dually targeted OE7 line (Figure 1 and Table 1) [4]. In this study, we investigated the impact on plant physiology of the overexpression of AtPAP2 solely in mitochondria (P2TOM lines) and solely in chloroplasts (P2TOC lines). Three time points of illumination (T = 0, 1, and 8) were investigated, as the activities of these two powerhouses behave differently under light and dark conditions.

The activity of photosynthesis can be evaluated by chlorophyll fluorescence parameters (Y(II), ETR, and qP) [32]. In our study, the ETR of OE7, P2TOC, WT, and P2TOM exhibited a descending order (Figure 3), suggesting that the overexpression of AtPAP2 in the chloroplasts of OE7 and P2TOC can speed up photosynthetic ETR, whereas the hyperactive mitochondria in P2TOM have a negative impact on photosynthetic ETR (Figure 3). NADPH and ATP produced from the light reaction are fed into the CBB cycle to fix carbon. Two of the 11 enzymes in the CBB cycle, NADP^+^-GAPDH and FBA, were reported to control the rate of carbon fixation in an upregulating manner [43,44]. Their capacity was found to be significantly increased in OE7 and P2TOC (GAPDH only) but decreased in P2TOM (Table 2). These data suggest that there is higher CBB capacity in the OE7 and P2TOC chloroplasts, thereby resulting in high sucrose (Figure 5) and high seed yield at maturity (Table 1). In contrast, lower chloroplastic activity but higher mitochondrial activity in P2TOM is consistent with the low sucrose (Figure 5) and low seed yield phenotypes of P2TOM (Table 1). The higher sucrose, faster plant growth, and higher seed yield phenotypes of the dually targeted OE7 line than the P2TOC line imply that the productivity of chloroplasts is highly dependent on mitochondrial activity. More active mitochondria in OE7 than P2TOC could indirectly regenerate more NADP^+^ in the chloroplasts for receiving electrons from the LEF.

During photosynthesis, sucrose is synthesized in the cytosol from the 3C compounds fixed by photosynthesis, and malate is generated from both organelles and accumulates in the vacuole [14,45]. Due to the inhibition of pyruvate dehydrogenase in the mitochondria under illumination, the conventional TCA cycle is restricted. The major source of reducing equivalents for mitochondrial ETC is thus generated from the glycine decarboxylase of the photorespiratory pathway, which exceeds the consumption capacity of the mitochondrial ETC, and the surplus reducing equivalents are exported in the form of malate to the cytosol via the malate–OAA shuttle [4]. By transferring electrons from the reducing equivalents along the ETC, a proton gradient is formed between the matrix and the intermembrane space to drive ATP synthesis [46]. Compared to WT, ROS signals were observed in the mitochondria of OE, P2TOC, and P2TOM lines after a short period of illumination (Figure 2), reflecting an overreduction of their ETC [8]. Taken together, these observations suggest that OE, P2TOC, and P2TOM have hyperactive mitochondria, reflected by the high ATP levels in the three AtPAP2 OE lines in the middle of the day (Figure 4). 

Under illumination, some of the malate synthesized in the mitochondria is converted to citrate, which is then exported and stored in the vacuole [47]. Citrate plays an important role in nitrogen assimilation and provides the backbone for amino acid synthesis [48,49]. At midday, both OE7 and P2TOM lines contained less citrate than WT, but higher levels of serine, tyrosine, and valine. Particularly, the P2TOM lines had significantly higher threonine and GABA than the other three lines at all three time points. These observations were not seen in the P2TOC line, except that tyrosine and valine were elevated in this line at midday (Figure 5). In all three OE lines, the accumulation of amino acids correlated with a lower citrate level than in WT at midday (Figure 5). In contrast, at the end of the night, OE7 had significantly higher citrate than WT, whereas P2TOM had significantly lower citrate. This is reasonable, as the OE7 line fixes more carbon but the hyperactive mitochondria of P2TOM consume more carbon or reducing equivalents.

In the dark, photosynthesis ceases and the TCA cycle operates in a cyclic mode to generate ATP at the expense of malate and carbohydrates. The mitochondria consume sugars released from starch metabolism in chloroplasts, the sucrose accumulated in the cytosol and vacuoles, and the reducing equivalents released from malate. It should be noted that OE7 and P2TOC accumulated higher sucrose and lower malate during photosynthesis (T = 8) (Figure 5), but their sucrose and malate contents were higher than WT in the dark, indicating that sucrose was preferably consumed over malate by the mitochondria of these two lines in the dark. P2TOM accumulated less sucrose than WT but significantly more ATP than WT during photosynthesis (Figure 4), indicating that its robust mitochondria are more active in ATP production. P2TOM also accumulated more serine and malate than P2TOC at midday, possibly due to a higher photorespiration rate (Figure 5). When sucrose is less abundant, P2TOM may use malate as the major source of reducing equivalents for feeding to the mitochondrial ETC, as the malate concentration dropped significantly during the night in the P2TOM line (Figure 5). 

Unlike the OE7 line, in the P2TOM line, due to its hyperactive mitochondria that consume more reducing equivalents without a concurrent enhancement in energy supply from chloroplasts, the energy balance between chloroplasts and mitochondria was affected. This led to higher ATP production at the expense of carbohydrate assimilation, which resulted in early senescence and lower seed yield at maturity. P2TOC with AtPAP2 solely overexpressed in the chloroplasts may fix more carbon and generate more reducing equivalents to fuel the mitochondria, resulting in high ATP and sucrose, and higher seed yield and ROS production. When chloroplastic and mitochondrial activities were simultaneously elevated in the OE7 line due to the overexpression of AtPAP2 on both organelles, OE7 could streamline the generation and consumption of reducing equivalents, and managed to generate higher levels of ATP and sucrose, leading to faster growth and high seed yield at maturity. These AtPAP2 OE lines (OE7, P2TOM, and P2TOC) are therefore excellent examples to illustrate how efficient cooperation between chloroplasts and mitochondria can boost plant growth and how imbalance in the production and consumption of reducing equivalents can affect plant physiology. 

## 5. Conclusions

Chloroplasts and mitochondria have been regarded as producers and consumers, respectively, of carbohydrates and reducing equivalents. The coordination between these two organelles in handling reducing equivalents is important for plant growth, as they streamline photosynthesis by balancing the stromal NADPH/ATP ratio and maintain plant health by preventing the overproduction of ROS. Here, our work provides real models to illustrate the importance of an optimized redox balance between chloroplasts and mitochondria for plant growth.

## Figures and Tables

**Table 1:** Flowering times and seed yields of WT and various OE lines under long-day regime.

Line	INF	NRF	Seed Weight per Plant (mg)	Yield Change (%)
WT	27.5 ± 0.9 ^a^	12.6 ± 0.9 ^a^	235.7 ± 31.9	-
OE7	20.4 ± 1.0 ^d^	6.8 ± 1.1 ^c^	331.6 ± 33.1 ^**^	+40.7
P2TOC1	25.8 ± 0.5 ^b^	11.4 ± 1.0 ^b^	292.7 ± 39.1 ^*^	+24.2
P2TOC2	25.6 ± 0.9 ^b^	11.6 ± 1.2 ^ab^	281.9 ± 28.6 ^*^	+19.6
P2TOM3	23.8 ± 1.3 ^c^	10.6 ± 1.3 ^b^	61.1 ± 15.4 ^**^	−74.1
P2TOM4	24.5 ± 0.6 ^c^	10.6 ± 0.8 ^b^	62.4 ± 31.4 ^**^	−73.5

For flowering time, mean values of 13–15 plants per line are shown. One-way ANOVA with Tukey’s honestly significant differences (HSD) test by PASW Statistics 18 software was used for statistical analysis; different letters (a, b, c, d) indicate significant differences (*p* < 0.05, *n* = 13–15). INF, average date of emergence of inflorescence; NRF, no. of rosette leaves on first day of appearance of inflorescence. Seeds were harvested from individual plants that were dried completely. Seed weights were recorded after drying seeds at 37 °C for 7 days. Data expressed as mean value of 6 plants per line (*n* = 6). Significant differences between transgenic lines and WT by t-test are indicated by asterisks (* *p* < 0.05, ** *p* < 0.001).

**Table 2:** Enzyme capacity of CBB and TCA cycles at midday.

Enzyme	Source	Enzyme Activity (nmol min^−1^ mg^−1^ Protein)			
		WT	OE7	P2TOM4	P2TOC1
FBA	Chloroplast	154 ± 0.6	180 ± 6.6 *	134 ± 4.8 *	158 ± 3.8
NADP^+^-GAPDH	Chloroplast	164 ± 5.6	176 ± 3.3 *	148 ± 4.5 *	183 ± 1.8 *
NADP^+^-MDH	Chloroplast	474 ± 4.5	552 ± 24 *	482 ± 37	584 ± 27 *
NAD^+^-MDH	Leaf	3273 ± 51	3289 ± 26	3790 ± 58 *	3511 ± 36 *
NAD^+^-MDH	Mitochondria	17,244 ± 130	18,144 ± 350 *	17,950 ± 80 *	17,455 ± 490
NAD^+^-ME	Mitochondria	137 ± 1.0	151 ± 1.7 *	162 ± 1.1*	149 ± 1.6 *
Citrate synthase	Mitochondria	5.4 ± 0.6	5.3 ± 0.9	5.5 ± 0.4	5.3 ± 0.0
Aconitase	Mitochondria	80.0 ± 4.6	79.1 ± 3.4	113.4 ± 1.7*	99.4 ± 0.3 *
NAD^+^-ICDH	Mitochondria	65.8 ± 1.7	84.8 ± 3.7 *	86.4 ± 1.4*	69.3 ± 0.9 *
NADP^+^-ICDH	Leaf	59.8 ± 1.7	41.3 ± 0.5 *	62.8 ± 1.7	61.9 ± 0.8
2OGDH	Mitochondria	18.1 ± 0.3	20.9 ± 0.5 *	19.5 ± 0.2*	18.3 ± 0.1 *
SDH	Mitochondria	63.1 ± 5.3	96.3 ± 8.0 *	76.4 ± 2.3*	83.0 ± 0.81 *
Fumarase	Mitochondria	440 ± 11	404 ± 15 *	441 ± 19	390 ± 12 *

Data are means ± SD of 3–4 biological replicates. Independent sample t-test was carried out. Significant differences between WT and either OE7, P2TOM4, or P2TOC1 are shown by asterisks. * *p* < 0.05, *n* ≥ 3. FBA, fructose bisphosphate aldolase; GAPDH, glyceraldehyde-3-phosphate dehydrogenase; ICDH, isocitrate dehydrogenase; MDH, malate dehydrogenase; ME, malic enzyme; 2OGDH, 2-oxoglutarate dehydrogenase; SDH, succinate dehydrogenase.

## Data Availability

The data supporting the findings of this study are available within the article and its supplementary materials.

## Supplementary Materials

The following are available online at https://www.mdpi.com/article/10.3390/antiox10060935/s1, Table S1. Vector constructs and primers in this study.
