# Supplementary material for: A Balance between the Activities of Chloroplasts and Mitochondria Is Crucial for Optimal Plant Growth
Source: Antioxidants (Basel). 2021 Jun 9;10(6):935. doi: 10.3390/antiox10060935 (PMC8228383; doi:10.3390/antiox10060935)
Supplement: Supplementary file 1 [file antioxidants-10-00935-s001.zip › antioxidants-1231047-supplementary.pdf]

Table S1. Vector constructs and primers in this study.

Vector	Primer Name	Primer Sequence (5′–3′)
**pCXSN_P2TOM /pCXSN_P2TOC**	AtPAP2-SalI-F	GGCCGTCGACATGATCGTTAATT TCTCTTTC
**pCXSN_P2TOM**	TOMoverlapF	GGCCGTCGACATGATCGTTAATT TCTCTTTC
**pCXSN_P2TOM**	P2overTOMR	ATCAGTGGGAGCAAAGTGAAGAATAAGAAAAG
**pCXSN_P2TOC**	TOCoverlapF	ATCAGTGGGAGCAAAGTAGATGGATCTTACTCT
**pCXSN_P2TOC**	P2overTOCR	GTAAGATCCATCTACTTTGCTCCCACTGATTAC
**pBI221**	TOM20-SpeI-GFPF	CGCAACTAGTGTGAAGAATAAGAAAAG
**pBI221**	TOM20-SacI-GFPR	TTTTGAGCTCTTAACGAGGAGGAGAGA
**pBI221**	TOC33-SpeI-GFPF	GCAGACTAGTGTAGATGGATCTTACTCT
**pBI221**	TOC33-SacI-GFPR	TTTTGAGCTCTTAAAGTGGCTTTCCAC
